# HumanNet v3: an improved database of human gene networks for disease research

**DOI:** 10.1093/nar/gkab1048

**Published:** 2021-11-08

**Authors:** Chan Yeong Kim, Seungbyn Baek, Junha Cha, Sunmo Yang, Eiru Kim, Edward M Marcotte, Traver Hart, Insuk Lee

**Affiliations:** Department of Biotechnology, College of Life Sciences and Biotechnology, Yonsei University, Seoul 03722, Korea; Department of Biotechnology, College of Life Sciences and Biotechnology, Yonsei University, Seoul 03722, Korea; Department of Biotechnology, College of Life Sciences and Biotechnology, Yonsei University, Seoul 03722, Korea; Department of Biotechnology, College of Life Sciences and Biotechnology, Yonsei University, Seoul 03722, Korea; Department of Bioinformatics and Computational Biology, University of Texas MD Anderson Cancer Center, Houston, TX 77030, USA; Center for Systems and Synthetic Biology, Institute for Cellular and Molecular Biology, University of Texas, Austin, TX 78712, USA; Department of Molecular Biosciences, University of Texas at Austin, TX 78712, USA; Department of Bioinformatics and Computational Biology, University of Texas MD Anderson Cancer Center, Houston, TX 77030, USA; Department of Biotechnology, College of Life Sciences and Biotechnology, Yonsei University, Seoul 03722, Korea

## Abstract

Network medicine has proven useful for dissecting genetic organization of complex human diseases. We have previously published HumanNet, an integrated network of human genes for disease studies. Since the release of the last version of HumanNet, many large-scale protein–protein interaction datasets have accumulated in public depositories. Additionally, the numbers of research papers and functional annotations for gene–phenotype associations have increased significantly. Therefore, updating HumanNet is a timely task for further improvement of network-based research into diseases. Here, we present HumanNet v3 (https://www.inetbio.org/humannet/, covering 99.8% of human protein coding genes) constructed by means of the expanded data with improved network inference algorithms. HumanNet v3 supports a three-tier model: HumanNet-PI (a protein–protein physical interaction network), HumanNet-FN (a functional gene network), and HumanNet-XC (a functional network extended by co-citation). Users can select a suitable tier of HumanNet for their study purpose. We showed that on disease gene predictions, HumanNet v3 outperforms both the previous HumanNet version and other integrated human gene networks. Furthermore, we demonstrated that HumanNet provides a feasible approach for selecting host genes likely to be associated with COVID-19.

## INTRODUCTION

Functional relations between highly wired genes underlie complex phenotypes of organisms. Hence, life scientists have tried to delineate interactions between genes and their products through diverse experimental and computational approaches. The integration of interactions inferred from various datasets and methods generally increases the reliability and coverage of a gene network (1). Many integrated human gene networks have been developed, and some are well maintained and used widely (2–6). We have also developed a functional network of human genes, HumanNet (7) by integrating inferred co-functional relations from diverse datasets, encompassing co-citation (CC) in PubMed articles, co-expression (CX), protein–protein interaction (PI), genetic interaction (GI), protein domain co-occurrence, and genomic context similarity. This network of human genes has been further expanded by including interactions between proteins evolutionarily conserved between human and other organisms. The initial version of HumanNet has gone through a major update via inclusion of functional omics data newly accumulated in public depositories. The resultant HumanNet v2 showed substantially improved performance on disease gene predictions (8).

Advanced high-throughput technologies accelerated data generation further and accumulated large amounts of functional 'omics data during the past few years. Therefore, updating HumanNet is a timely task for further improvement of network-based predictions of disease genes. Here, we present HumanNet v3 (https://www.inetbio.org/humannet/), which showed a significant performance improvement because of the inclusion of the new data and better network inference algorithms. HumanNet v3 supports a three-tier model: HumanNet-PI (a protein–protein physical interaction network), HumanNet-FN (a functional gene network), and HumanNet-XC (a functional network extended by CC). Users can select an appropriate tier of the network for their purpose of a study. Using multiple sources of disease gene annotations, we proved that HumanNet v3 outperforms HumanNet v2 and other integrated networks of human genes. In addition, we demonstrated that HumanNet-based prediction can prioritize human genes that are highly likely to be associated with COVID-19, suggesting its usefulness in COVID-19 research.

## EXPANSION OF HUMANNET

### Improvements in network inference

**Figure 1.**
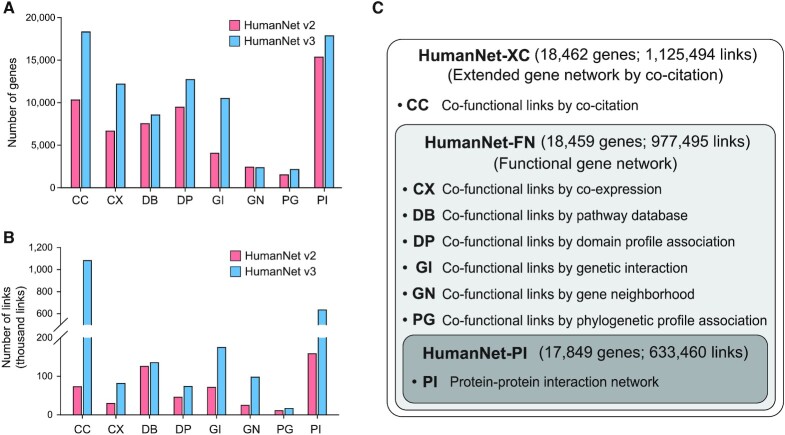
An overview of HumanNet v3. (A, B) Bar graphs illustrating improvements in the numbers of genes (**A**) and functional links (**B**) as compared to HumanNet v2. (**C**) A summary of the three-tier model of HumanNet v3.

Detailed descriptions of the network construction are provided in Supplementary Methods, and the improvements of HumanNet v3 compared with HumanNet v2 are summarized in Tables 1. Here, we briefly describe the updates in methods and data sources. We first updated gold standard gene pairs based on shared pathway annotation because their size and quality are critical for network model training and evaluation. We generated a set of gold standard gene pairs using the latest release of Gene Ontology Biological Process (GOBP) (2021-03-08 release) (9) and MetaCyc (release 22.5) (10). For GOBP, we used only IDA and IMP evidence codes to generate reliable gold standard gene pairs. As a result, the number of gold standard gene pairs almost doubled (from 124 950 to 260 962 links) while genome coverage increased by 69% (from 5190 to 8779 genes) as compared to the previous version. Size expansion of training data generally improves network modeling, often by salvaging functional links that were excluded due to under-evaluation because of insufficient size of the previous training data. For example, HumanNet v3 inherited the inferred functional links based on CX and phylogenetic profiling (PG) from the previous HumanNet but rescued substantially more links with higher likelihood than that of random gene pairs by retraining them with the new gold standard data (Figure 1A, B, Supplementary Table S1, Supplementary Figure S1). All network links were evaluated by means of a log likelihood score scheme, just as was the case for the earlier HumanNet.

**Table 1.** Comparison between HumanNet v2 and v3

**Component network**	HumanNet v2	HumanNet v3
**Gold Standard**	Gene Ontology Biological Process (21 October 2012) (IDA, IMP); MetaCyc	Gene Ontology Biological Process (8 March 2021) (IDA, IMP); MetaCyc r22.5
**CC**	Based on ∼300k full-text articles from PubMed Central	Based on ∼650k full-text articles from PubMed Central; Updated algorithm for link prioritization
**CX**	Based on 125 microarray-based and 33 RNA-seq-based GSEs (16,220 samples in total)	Inherited from HumanNet v2; Re-trained with the new Gold Standard
**CE → GI**	Co-essentiality links based on >100 shRNA and > 400 CRISPR-Cas9-based essential gene profiles	Genetic interactions from BioGRID and iRefIndex r14 and co-essentiality links based on ∼800 CRISPR Cas9-based essential gene profiles
**DB**	Based on three pathway databases [KEGG (5 January 2017), BioCarta (5 January 2017), and Reactome (3 January 2017)]	Latest version of the databases [KEGG (12 April 2021), BioCarta (12 April 2021), and Reactome (14 April 2021)]; Updated algorithm for link prioritization
**DP**	Based on domain profiles by InterPro r46 Profile	Based on domain profiles by InterPro r84 Profile
**GN**	Based on 1748 prokaryotic (1626 bacteria and 122 archaea) genomes, 754 human metagenomes and 242 ocean sample metagenomes	Based on 9428 genus representative genomes of Prokaryotes from GTDB r95
**IL**	Transfer 10 latest functional gene networks for five species and transfer PIs of four vertebrate species (dog, cattle, rat and chicken) in iRefIndex r14; All orthology-transferred networks were integrated into a single network	Inherited from HumanNet v2; Excluded from the final HumanNet v3
**PG**	Based on 1626 bacterial and 122 archaeal genomes Analyzed two phylogenetic profiles for bacteria and Archaea, separately.	Inherited from HumanNet v2 Re-trained with the new Gold Standard
**LC → PI**	Non-redundant PI set from IRefIndex r14	Non-redundant PI set from iRefIndex r17, BioPlex1, 2 and 3, BioGRID (v4.3.196), and IntAct (10 March 2021) databases; updated algorithm for link prioritization
**HT → PI**	Based on seven protein complex mapping data sets and five binary PI screen data sets	

CC: co-citation; CX: co-expression; CE: co-essentiality; GI: genetic interaction; DB: database; DP: domain profile; GN: gene neighboring; IL: interolog; PG: phylogenetic profile; LC: literature curation; HT: high-throughput protein–protein interaction; PI: protein–protein interaction.

We also improved HumanNet by expanding the source data and optimizing the network inference algorithms. The number of full-text PubMed Central articles used for constructing the CC network grew from 293 139 to 694 572, a 2.37-fold increase. In addition, there have been considerable updates of human PI maps as a consequence of large-scale experiments (11–13). Therefore, we updated the PI data using the non-redundant set of the latest IntAct (14), iRefIndex (15), BioGRID (16) and BioPlex (12). Furthermore, we took full advantage of updated pathway databases such as KEGG (17), Reactome (18) and Biocarta (19) to infer functional links by means of the pathway database (DB). We have previously prioritized the links for CC, PI and DB networks with accounting for the specificity of interactions (i.e. links for hub genes received less weight). Nevertheless, for the previous HumanNet, we did not use the number of pieces of supporting evidence for each pair of functionally associated genes. For HumanNet v3, we gave more weight to gene pairs with more pieces of supporting evidence for the associations (Supplementary Figure S2). We observed a substantial increase in genome coverage and sizes of CC, PI and DB networks as a result of the expanded source data and edge scoring with taking account of all supporting evidence for each gene pair (Figure 1A, B, Supplementary Table S1). In particular, the network size increased ∼15-fold (from 72 819 to 1 081 518) and ∼4-fold (from 158 499 to 633 460) for the CC and PI network, respectively.

Co-functional networks based on a gene neighborhood (GN), domain profile association (DP), and GI were also improved by the enlarged data sources. Recently, the phylogenetic tree of prokaryotic species was expanded significantly by the addition of species identified by means of metagenome-assembled genomes (20,21). HumanNet v3 takes advantage of the GN relations in newly identified prokaryotic species’ genomes available in the Genome Taxonomy Database (GTDB) (22). The functional associations mediated by a GN were inferred from 9428 prokaryotic genomes for HumanNet v3, whereas HumanNet v2 involved fully sequenced genomes only from 1746 prokaryotes and 996 metagenomic contigs. Consequently, the GN network size increased ∼4-fold (from 24 862 to 97 565; Figure 1B). The inference of the domain profile association network is also based on updated InterPro (release 84) (23). Although we employed the same weighted mutual information scheme (24) as in the previous HumanNet, we obtained ∼60% more links than in the previous network (an increase from 45 958 to 73 414). HumanNet v2 contains a co-essentiality network, which is a type of GI network inferred from large-scale CRISPR–Cas9 knockout profile similarities between genes. We combined GIs based on small-scale knockout assays retrieved from databases with the coessentiality network to construct a GI network for HumanNet v3. We retrieved GIs from BioGRID (16) and iRefIndex (15) and inferred co-essentiality links from updated DepMap (25) (2020 Q4 version). Consequently, the GI network size for HumanNet v3 increased ∼2.5-fold (from 71 243 to 174 509) (Figure 1B).

### Interologs decreases network accuracy for human disease genes

Interologs (26), protein-protein interactions transferred from other species via orthology, can also map functional associations between genes of a target species. Many integrated gene networks include interologs because they often improve network coverage and prediction performance, especially when there are difficulties in obtaining sufficient edge information directly from the target species genes (27). Nonetheless, given that many small- and large-scale protein-protein interaction mapping projects for human genes were carried out in the past several years, interologs from other species may no longer supplement human interactome information. To test this hypothesis, we obtained human interologs and integrated them with all eight component networks described above (CC, CX, DB, DP, GI, GN, PG and PI). We then investigated whether the incorporation of the interologs improves accuracy of connecting genes for the same diseases by GWAS Catalog (28) or DisGeNET (29). We found that the incorporation of interologs decreases network accuracy for human disease genes (Supplementary Figure S3). These results suggest that the current human interactome already covers most of evolutionarily conserved protein-protein interactions, and additional interactions transferred from other species via orthology may introduce more false positives than true associations between human disease genes. On the basis of these results, we finally decided not to include interologs in HumanNet v3.

### The three-tier model of HumanNet v3

Users may benefit from a network more appropriate for their research purpose. For example, a protein–protein physical interaction network will be more suitable for mode-of-action studies of disease mutations, and a network with no CC links may reduce over-evaluation of disease gene prediction by the literature bias toward disease studies. The previous HumanNet was based on four tiers including networks that contain interologs. As interologs are no longer incorporated into HumanNet, we offer a three-tier model for HumanNet v3: HumanNet-PI (protein-protein interaction network), HumanNet-FN (functional gene network), and HumanNet-XC (gene network extended by CC) (Figure 1C). HumanNet-PI consists of physical interactions only. Therefore, users who wish to study protein complexes or ligand–receptor interactions may utilize HumanNet-PI. HumanNet-FN is an integrated functional gene network encompassing associations between human genes derived via diverse computational and experimental approaches, including a PI network. HumanNet-XC is a gene network further expanded by CC links. Although a CC network could substantially increase network coverage and prediction performance, it may face circular reasoning when network performance is evaluated by means of literature-based resources. We recommend HumanNet-XC to users who want to exploit the full prediction power of HumanNet whereas HumanNet-FN to those who need a more conservative analysis.

The increases of each component network led to significant expansion of the integrated networks. The most inclusive network, HumanNet-XC, contains 1 125 494 links, which is more than twice the number of links for the largest HumanNet v2 (525 537 links). The coverage of the human protein coding genome also increased from 17 929 in HumanNet v2 to 18 462 genes in HumanNet v3, thereby covering 99.8% of protein coding genes of the consensus coding sequence (CCDS) r22 (30).

### Assessment of HumanNet v3 for disease gene prediction

Next, we systematically evaluated HumanNet v3 for disease gene predictions. We compared the previous HumanNet (v2) with full size (XN) (8) and the three tiers of HumanNet v3: HumanNet-PI, FN and XC. We also assessed other publicly available integrated human gene networks such as STRING (v11.5) (2), GeneMania (as of 27 April 2021) (5), ConsensusPathDB (as of 31 July 2021) (4), PCNet (as of 5 August 2021) (31), FunCoup (v5.0) (3) and GIANT (as of 25 September 2021) (6) (Supplementary Table S2). Similar to HumanNet, these networks consist of functional associations derived from diverse evidence. First, network precision was tested based on the proportion of gene pairs that share the same disease annotations. Except for PCNet, all the networks have edge scores; thus, the network precision levels were measured with the cost of disease genome coverage. Two independent databases of disease gene annotations—GWAS Catalog (28) and DisGeNET (29)—were employed to benchmark disease gene predictions. To conduct a more conservative evaluation, we removed GWAS candidate genes from the articles that were used for our inference of the CX network. This modification of the benchmarking dataset may reduce the chances of circular reasoning in disease gene predictions. Likewise, we used only a curated set of disease–gene associations from the DisGeNET database to reduce bias toward literature-based gene interactions.

**Figure 2.**
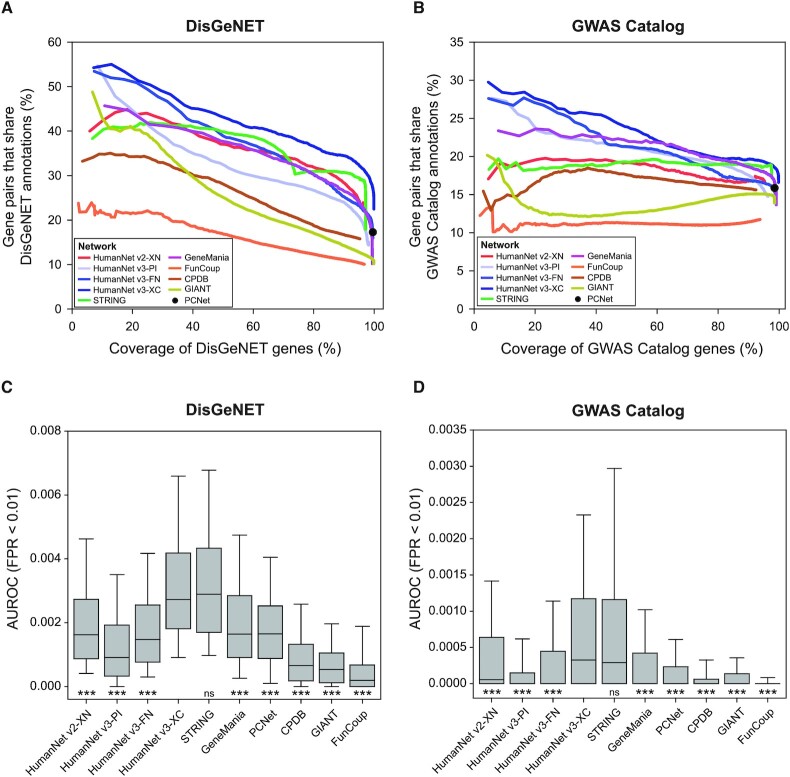
Network assessment for disease gene predictions. (**A**, **B**) The percentage of gene pairs that share disease annotation (y-axis, link precision) according to the DisGeNET (A) and GWAS Catalog (B) gene coverage (x-axis, gene recall) are cumulatively calculated for every 1000 links from the top links. As the PCNet network has no link score, the link precision and gene recall are calculated for the entire link. (**C**, **D**) The area under the receiver-operating characteristic curve (AUROC) up to a false positive rate (FPR) of 1% was measured for the network-based retrieval of disease genes annotated by DisGeNET (C) or GWAS Catalog (D) (***P < 0.0001, ns: *P* > 0.05 according to the two-tailed Mann–Whitney *U* test).

We first measured the percentage of links that shared the same disease annotations and the coverage for the disease gene set. For DisGeNET disease genes, among the three tiers of HumanNet v3, HumanNet-XC showed the highest precision, followed by HumanNet-FN and HumanNet-PI (Figure 2A). Compared to HumanNet v2, HumanNet-XC manifested clearly higher precision across the entire range of disease genome coverage, and HumanNet-FN showed comparable precision. Notably, HumanNet-XC outperformed all other integrated human gene networks across the entire range of disease genome coverage. For the disease genes from GWAS Catalog, we observed a similar ranking of network precision among the networks (Figure 2B). Taken together, these results indicate that disease gene connections identified by HumanNet v3 are generally more accurate than those inferred by the previous HumanNet and by other integrated human gene networks available publicly.

Network accuracy contributes to the performance of a network-based disease gene prediction. Here, we evaluated disease gene prediction via direct neighbors in the networks. Conceptually, genes that connect to a group of disease genes are likely to be involved in the same disease. Indeed, genes known for the same disease tend to be connected with each other. Thus, if we prioritize genes on the basis of the connectivity, the genes known to be involved in a disease are expected to be retrieved with high rankings. The retrieval rate of known disease genes can be measured by receiver-operating characteristic (ROC) analysis, and its results can be summarized as the area under the ROC curve (AUROC). Because we generally consider only the top hundreds of candidates for follow-up functional analysis, the AUROC for early retrieval is practically more important. Therefore, we assessed the disease predictions based on the AUROC up to a false positive rate of 1%. We found that HumanNet-XC and STRING share the first place in terms of performance among all the tested networks for the comparison with disease genes annotated by both DisGeNET and GWAS Catalog (Figure 2C, D). Disease gene prediction by HumanNet-XC was not significantly different from that of STRING but significantly outperformed all the other networks (*P* < 0.0001, two-tailed Mann–Whitney *U* test). Because network size also contributes to network-based gene prioritization, STRING may compensate for its lower network precision (as compared to HumanNet-XC, see Figure 2A, B) by ∼5-fold more links (Supplementary Table S2). Overall, we conclude that HumanNet v3 and STRING (separately) perform best on disease gene prediction.

## UPDATES IN THE WEB INTERFACE

The HumanNet v3 web server largely inherited the user interface from the previous version. On the other hand, there are major improvements in network information. The most important update is the inclusion of literature data from PubMed that support PI and CC associations. In addition, all disease annotations were updated by means of the latest versions.

We continue to offer two prediction modules on the HumanNet v3 web server: network-based disease gene prediction and network-based disease annotation prediction. In the network-based disease gene prediction, a user submits a known gene set, dubbed guide genes, for a disease of interest. Then, the user can examine network neighbors of the guide genes in a network view. The network view is implemented interactively; accordingly, the user can obtain detailed information on a link or gene of interest by selecting an object. In HumanNet v3, we reinforced the link information by presenting supporting evidence (publications) for PI and CC links. Next, the web server outputs metrics of how much the guide genes are interconnected in the network and predictive power of HumanNet for the guide genes according to ROC analysis. The web server also presents candidate genes for the given diseases; the genes are sorted by network prioritization scores. In network-based disease annotation prediction modules, a user submits a query gene(s). After that, the web server predicts the diseases potentially associated with the query gene by retrieving disease annotations from its neighbors. HumanNet v3 web server provides the latest versions of five gene annotation databases for pre-defined guide genes and prediction interpretation: GOBP (9), GWAS Catalog (28), DisGeNET (29), DISEASES (32) and Human Phenotype Ontology (33) (Supplementary Table S3). Detailed methods and interpretations for the two disease research modules are described on the ‘Tutorial’ tab of the web server. Finally, on the ‘Download’ tab of the web server, we provide gold standard gene pairs and all the component networks that were integrated into HumanNet v3.

## CASE STUDY: HUMANNET-BASED PREDICTION OF HOST GENES FOR COVID-19

**Figure 3.**
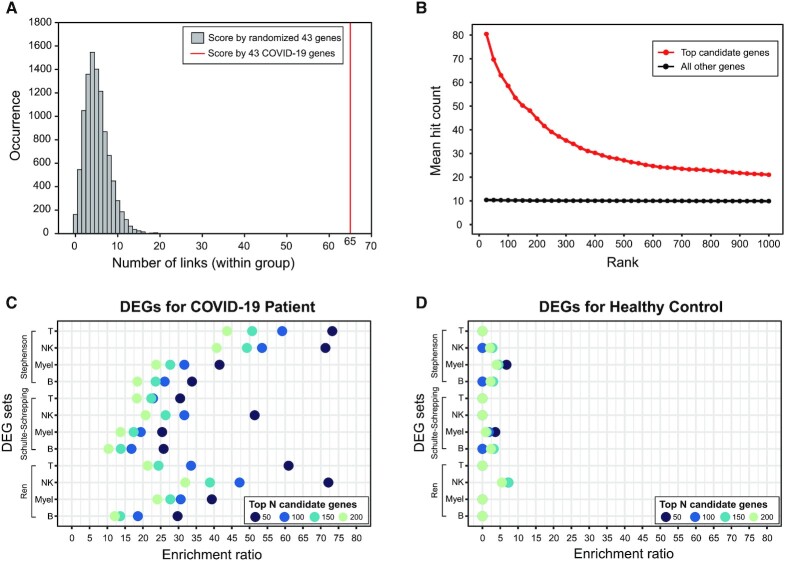
Validation of HumanNet-based candidate genes for COVID-19. (**A**) The number of connections between 43 guide genes derived from COVID-19 genome-wide association studies (GWAS) in HumanNet-XC. The histogram represents the distribution of network connectivity from 10 000 random 43 genes, and red vertical line indicates the number of connections between the 43 guide genes. (**B**) Mean hit count to 722 COVID-19 related gene sets. The red line and black line represent the mean hit count for top candidates and that for all other genes, respectively. (C-D) Enrichment ratio of DEGs specific for COVID-19 patients (**C**) and healthy controls (**D**) among top candidate genes. Different size of top candidates for validation were marked by color codes. DEGs were derived from three independent studies (Stephenson *et al.* (36), Schulte-Schrepping *et al.* (37), and Ren *et al.* (38)) and four distinct cell types (T, T cells; NK, natural killer cells; Myel, myeloid cells; B, B cells).

COVID-19 is a highly contagious disease by severe acute respiratory syndrome coronavirus (SARS-CoV-2), resulting in more than 4.7 million deaths worldwide as of September 2021 (https://covid19.who.int/). Many human genes are involved in infectious diseases. Therefore, identification of host genes associated with COVID-19 will facilitate development of strategies for its prevention and treatment. To demonstrate the utility of HumanNet in COVID-19 study, we performed web-based disease gene prediction with guide genes that are known to be associated with COVID-19. Recently, international consortium of COVID-19 Host Genetics Initiative published results from genome-wide association studies (GWAS) comprised >49 000 patients (34). We compiled 43 human genes reported by the COVID-19 GWAS (Supplementary Table S4, Supplementary Methods) and submitted them into HumanNet web server. We found that the 43 guide genes are significantly more connected to one another in the HumanNet-XC compared to the same size of randomized gene sets (Figure 3A, *P* < 0.0001 by permutation test). HumanNet-XC prioritized a total of 4418 candidate genes by connection to the 43 guide genes.

To confirm the validity of the network-based gene prediction, we utilized a community-wide collection of 722 COVID-19 related gene sets (35) derived from various experimental assays such as genome-wide CRISPR screens, genome-wide differential expression analysis in cells and tissues, and physical interactions with SARS-CoV-2 proteins. Genes that are likely to be associated with COVID-19 will appear among the hit lists of many experimental studies. Accordingly, we may expect to observe larger hit count to the 772 gene sets for more likely COVID-19 genes. Hit counts of human genes for the 772 gene sets are summarized in Supplementary Table S5. We validated HumanNet-based predictions for COVID-19 genes based on mean hit count of candidate genes (Supplementary Methods). The genes in top 50 ranks showed ∼7-fold higher mean hit count compared to that for all other genes. The mean hit count decreases as rank index increases and maintained ∼2-fold of that for all other genes after top 500 ranks (Figure 3B). These results suggest that top candidate genes by HumanNet v3 are highly likely to be involved in COVID-19. We also validated predicted genes by differentially expressed genes (DEGs) for COVID-19 patients or healthy controls generated from three independent single-cell RNA sequencing studies (36–38). For each study, we compiled DEGs from four immune cell types: T cells, B cells, natural killer cells and myeloid cells (Supplementary Methods, Supplementary Table S6). We calculated enrichment ratio of the proportion of DEGs for top-ranked genes compared to that for all other genes. Across all cell types, top-ranked genes were more enriched for DEGs from COVID-19 patients than DEGs from healthy controls. Furthermore, more highly-ranked genes resulted in higher enrichment ratio of COVID-19 specific DEGs (Figure 3C, D). These results demonstrated feasibility of HumanNet-based gene prioritization for COVID-19. This case study could be easily reproduced in the HumanNet web server.

## CONCLUSIONS

In this report, we present an updated HumanNet version with major expansion of network information. Interologs were not integrated into HumanNet v3 because we noticed a decrease in the accuracy of the network for disease gene associations. This finding implies that protein-protein interaction mapping during the past few years filled out a large portion of the human interactome, which has been supplemented by network information transferred from other species only. Despite the exclusion of interologs, we expanded HumanNet by more than twofold as compared to the previous version. We found that HumanNet v3 outperforms both the previous HumanNet and most of the integrated human gene networks currently available publicly. Finally, we demonstrated that HumanNet can predict host genes associated with COVID-19. These results together support that the improved HumanNet will continue to provide an effective resource for the study of a wide variety of human diseases.

## DATA AVAILABILITY

HumanNet v3 is available under the Creative Commons Attribution-ShareAlike 4.0 International License at https://www.inetbio.org/humannet. The data can be accessed through the following web browsers: Google Chrome, Microsoft Edge, Apple Safari, Mozilla Firefox.

## Supplementary Material

gkab1048_Supplemental_Files

## Notes

Present address: Eiru Kim, Genomics Institute of Novartis Research Foundation, San Diego, CA, USA.
