# Supplementary material for: HumanNet v3: an improved database of human gene networks for disease research
Source: Nucleic Acids Res. 2021 Nov 8;50(D1):D632–9. doi: 10.1093/nar/gkab1048 (PMC8728227; doi:10.1093/nar/gkab1048)
Supplement: gkab1048_Supplemental_Files [file gkab1048_supplemental_files.zip › HumanNet_v3_Supplementary Infomation.pdf]

Supplementary Information

HumanNet v3: An improved database of human gene networks for disease research

Chan Yeong Kim, Seungbyn Baek, Junha Cha, Sunmo Yang, Eiru Kim, Edward M. Marcotte, Traver Hart, and Insuk Lee

Supplementary Methods

Human genes and gold standard gene pairs for network construction

We constructed HumanNet with 18,627 high-confidence human coding genes provided by the consensus coding sequence (CCDS) r22 (1). All the analyses in this study were based on the 18,627 genes. To construct gold standard positive gene pairs for network training and evaluation, we used Gene Ontology Biological Process (GOBP) (as of March 8, 2021) (2) and MetaCyc (release 22.5) (3) pathway annotations. For GOBP, we used only IDA and IMP evidence codes to generate reliable gold standard gene pairs. Genes within the same pathway were paired and considered as gold standard positive gene pairs for functional gene network. Next, gold standard negative gene pairs were generated by pairing the genes annotated by the GOBP or MetaCyc but not sharing any pathway annotation. To avoid bias toward a few pathways with many member genes, we disregard pathways with more than 100 member genes. Similarly, we used tier 1 data (literature curation by scientists) of MetaCyc only to achieve high reliability of the gold standard data. We also removed super-pathways to avoid bias toward inclusive pathway concepts. As a result, we obtained 260,962 gold standard positive gene pairs among the 8,779 human genes.

Bayesian framework for benchmarking and integrating HumanNet

We constructed HumanNet with supervised machine learning techniques validated previously (4-6). For the prioritized gene pairs inferred from given data (*D*), we calculated log-likelihood score (*LLS*) for every 1,000 links using a Bayesian statistic framework with the following equation:

$$LLS = \ln\left(\frac{P(L|D)/P(\neg L|D)}{P(L)/P(\neg L)}\right)$$

, where $P(L|D)$ and $P(\neg L|D)$ account for the probability of positive and negative gold standard gene pairs in given dataset. $P(L)$ and $P(\neg L)$ represent the probability of gold standard positive and negative links, respectively.

Next, we integrated the network with the weighted sum (*WS*) method as described in previous studies (4-6). For the co-functional gene pairs that are supported by the multiple datasets, the *WS* score is obtained with the following equation:

$$WS = LLS_0 + \sum_{i=1}^{n} \frac{LLS_i}{W \times i}\ , for\ all\ LLS\ \geq T,$$

, where LLS_0 indicates the maximum LLS for the gene pairs and LLS_i are sorted LLS scores by decreasing order. The weight factor, *W*, and *LLS* threshold, *T*, are optimized for maximizing the area under the plot of *LLS* versus gene coverage.

Construction of co-citation (CC), protein-protein interaction (PI), and pathway database (DB) network

Genes that frequently appears in the same biomedical papers tend to be functionally coupled. We inferred co-citation associations between human genes as for the previous HumanNet (5). Briefly, we constructed a co-citation network by searching official symbols of human genes from 647,572 PubMed Central full-text articles with the "human" Medical Subject Headings (MeSH) term (as of April 20, 2021). This was over twice number of the articles used for HumanNet v2 co-citation network (293,139).

To expand the protein-protein interactions (PPIs) of the HumanNet, we collected a non-redundant set of IntAct (as of March 10, 2021) (7), BioGRID (version 4.3.196) (8), and iRefindex (release 17) (9). We used the interactions with MI ontology (10) under the "MI:0915 physical association" to obtain PPIs only. We also compiled the recently published PPIs by large-scale experiment from BioPlex3 (11). We considered the protein complex with obvious bait-prey annotation to generate links by spoke-model which takes bait-prey links only.

If a pair of genes participate in the same pathways, they might be functionally associated. We downloaded pathway annotations from the latest KEGG (as of April 12, 2021) (12), BioCarta (as of April 12, 2021) (13), and Reactome (as of April 14, 2021) (14) databases. For the KEGG database, we excluded functional terms that belong to "Global and overview maps" and the terms with more than 400 annotated genes. Likewise, we used the lowest level functional terms and TAS (traceable author statements) evidence from Reactome annotations.

We prioritized the interactions of the CC, PI, DB networks based on the significance of the association by Fisher's exact test. In HumanNet v3, we consider the number of evidence-link to generate a contingency table, whereas the previous version only considers link counts (**Supplementary Figure 2A**). This change eventually weighs more on the association supported by more evidence. For the CC and PI network, the evidence corresponds to the supporting paper (by PMID) of the link. Likewise, the evidence corresponds to the supporting pathway terms of the link for the DB network. We could obtain a higher-scored and better-prioritized network with the revised method (**Supplementary Figure 2B-C**).

Construction of genetic interaction (GI) network

We obtained genetic interactions from iRefIndex (release 17) (9) and BioGRID (v4.3.196) (8). We filtered genetic interaction links by MI ontology terms of MI:0208, MI:0935, MI:2368, MI:2369, MI:2370, MI:2371, MI:2373, MI:2374, MI:2375, MI:2376, MI:2377, and MI:2378. These interactions were scored by the *LLS* scheme. We also inferred co-functional interactions based on ~800 CRISPR pooled library screens downloaded from DepMap (15) 2020Q4 version. We processed raw read counts to get essentiality scores (Bayes Factor, BF) using the best practice pipeline of BAGEL2 (16). Unlike the previous version of co-essentiality network, we did not filter out genes based on their essentiality. Quantile normalization was performed against all cell lines to reduce batch effect. Then, we calculated Pearson correlation coefficient (*PCC*) of any possible combination of two genes to measure co-essentiality across screens from various contexts. We benchmarked interactions using conditional *LLS* scheme described in Lawson *et al*. (17). Total of ~183,000 gene-gene interactions were obtained. The final GI network was constructed by integration of the scored genetic interactions from databases and co-essentiality network using the weighted sum method.

Construction of gene neighborhood (GN) network

Genes that are involved in the same biological function are frequently located adjacently in prokaryotic genomes. Gene neighboring networks infer a functional association between human genes when their prokaryotic orthologs are located close to each other (18). Recently, the phylogenetic diversity of uncultured prokaryotes has increased significantly by the incorporation of the metagenome-assembled genomes. To fully exploit the newly identified prokaryotic species, we used genomes from Genome Taxonomy Database (GTDB) (19) as a reference for constructing a gene neighborhood network. We found that the number of species differs widely across genera. For instance, *Streptomyces* genus contains 571 species, whereas the average number of species per genus was three. Therefore, we selected a genome with the highest completeness for each genus, resulting in 9,428 genus representative genomes. We then inferred probability- and distance-based gene neighborhood networks (20) based on the 9,428 genus representative genomes and integrated them into a final gene neighborhood (GN) network for HumanNet v3.

Construction of domain profile (DP) network

The protein domain is a functional unit of the protein; therefore, similar domains shared by a couple of genes could imply their functional associations (21). Domain information for human proteins was obtained from the latest InterPro (release 84) database (22). Similar to HumanNet v2, we deployed weighted mutual information (WMI) scoring scheme to construct the DP network (23). WMI method could infer stronger functional association by weighing more on rare protein domains.

Rescoring networks inherited from the HumanNet v2: co-expression (CX), phylogenetic profiling (PG), and interolog networks

For the CX, PG, and interolog networks, we used raw data from the HumanNet v2. Briefly, the CX network was constructed by integrating the sub-co-expression networks derived from the 158 GEO series. Phylogenetic profile was calculated based on the 1,626, 122, and 396 fully sequenced bacterial, archaeal, and eukaryotic genomes, respectively. Although the raw data resources were the same as the previous version, we could incorporate substantially more functional links into the

HumanNet v3 by rescoring the networks with the newly generated gold standard gene pairs. Indeed, the networks evaluated by the new gold standard showed increased *LLS* and fit better to the regression models compared to the original networks scored by HumanNet v2 gold standard (**Supplementary Figure 1**). As a result, we could take more links and nodes for the HumanNet v3.

The interologs are no longer included in the HumanNet v3. However, to test whether interologs improve the performance of the network, we constructed an interolog network with HumanNet v2 raw dataset. Interolog network was constructed by transferring protein-protein interaction networks from nine model organisms: *S. cerevisiae, M. musculus, D. rerio, D. melanogaster, C. elegans, C. l. familiaris, B. taurus, R. rattus,* and *G. gallus*. The networks of the model organisms were transferred to human orthologs by using the inparanoid algorithm. Similar to the PG and CX network, we retrained the network with HumanNet v3 gold standard. Finally, we obtained 122,697 functional associations between 9,001 human genes.

Assessment of networks for disease gene prediction

To compare the network performance in disease gene prediction, we compiled five publicly available human gene networks: STRING (v11.5) (24), FunCoup (v5.0) (25), GeneMania (as of April 27, 2021) (26), GIANT (as of September 25, 2021) (27), ConsensusPathDB (CPDB) (as of July 31, 2021) (28), and PCNet (as of August 5, 2021) (29) (**Supplementary Table 2**). We first filtered the networks for the 18,627 CCDS genes. For duplicate links, we only take the link with a higher score. CPDB network contains both scored and non-scored associations. Therefore, we assessed CPDB with both scored and non-scored versions and presented favorable results. As a reference for the network assessment, we gathered disease-gene associations from the GWAS Catalog (30) and DisGeNET databases (31). To avoid the bias toward co-citation networks by circular logic, we exclude GWAS Catalog disease-gene associations from the publications used to construct the co-citation network. In the DisGeNET associations, we only used a curated dataset.

Network performance was assessed by two metrics: precision of the link against gene coverage and prediction performance of the disease genes. For the link precision assessment, the cumulative percentage of the link that shares disease annotations was calculated for every 1,000 links starting from the highest confidence score. We also calculated the cumulative gene coverage for the disease

genes and plotted against the link precision. PCNet is a qualitative network with no edge score. Therefore, we simply calculated the precision and coverage for the entire network. Network-based disease gene predictions were performed by propagating disease information to direct neighbors only. We then conducted receiver operating characteristic (ROC) analysis to evaluate the retrieval rate of known disease genes. Finally, we compared the network performance using the area under the ROC curve (AUROC) until the false positive rate of 1%.

HumanNet-based prediction of host genes associated with COVID-19

We obtained author-reported 47 genes associated with COVID-19 from multi-cohort meta-analysis of genome-wide association studies comprised more than 49,000 patients (32). Because HumanNet is based on CCDS genes, we used only 43 genes defined by CCDS out of the 47 genes for HumanNet-based disease gene prediction (**Supplementary Table 4**). We performed network-based disease gene prediction via HumanNet web server with the 43 genes as guide genes. We obtained 4,418 candidate genes connected to the 43 guide genes with HumanNet-XC. These candidate genes were ranked by sum of LLS to the 43 guide genes.

Validation of top candidates by hit count for public COVID-19 gene sets and enrichment of DEGs for COVID-19 patients

We downloaded 722 gene sets related with COVID-19 available from https://maayanlab.cloud/covid19/ (as of September 6, 2021) (33). These gene sets were collected from various experimental assays such as genome-wide CRISPR screens, genome-wide differential expression of genes in cells and tissues, and physical interaction with SARS-CoV-2 proteins. Genes that are highly likely to be associated with COVID-19 will appear among the hit lists from many experimental studies. Hit counts for 772 gene sets are summarized in **Supplementary Table 5**. We calculated mean hit counts for a group of top candidate genes and that for a group of all other human genes with increment of 25 genes.

In addition to the public GOVID-19 gene sets, we also generated in-house validation gene sets based on differentially expressed genes (DEGs) for COVID-19 patients or healthy controls identified from three independent single-cell RNA sequencing (scRNA-seq) studies. With peripheral blood mononuclear cells (PBMCs) scRNA-seq data published by Stephenson et al. (34),

we used all COVID-19 patients except ones with asymptomatic conditions. For healthy controls, we used control patients labeled as healthy. From a large-scale atlas data published by Ren et al. (35), only PBMC scRNA-seq data were used for DEG analysis. For this data set, we divided COVID-19 patients into two groups, mild/moderate and severe/critical symptoms, and compared them to healthy controls, separately. For dual-center study data published by Schulte-Schrepping et al. (36), we used Cohort 2 PBMC scRNA-seq data that were generated by BD Rhapsody system. As for other data, we selected two groups of COVID-19 patients with severe and mild symptom and that of healthy controls. For all three studies, only cells annotated by authors as one of four major cell types (T cells, B cells, natural killer cells, myeloid cells) were used for generation of gene sets. We identified DEGs with the function FindMarkers() from Seurat v4.0.2 with R version 4.0.3. We used default parameters for the function except using natural logarithm for log fold-change. By comparing COVID-19 patients to healthy controls for the same cell type, DEGs with log fold-change > 0.25 and adjusted p-value < 0.01 were selected for each validation gene set. DEGs from the three scRNA-seq studies are summarized in **Supplementary Table 6**. Enrichment ratio was determined by dividing the proportion of DEGs for top N (50, 100, 150, 200) candidate genes by the proportion of DEGs for all other human genes.

Supplementary References

1. Pujar, S., O'Leary, N.A., Farrell, C.M., Loveland, J.E., Mudge, J.M., Wallin, C., Giron, C.G., Diekhans, M., Barnes, I., Bennett, R. *et al.* (2018) Consensus coding sequence (CCDS) database: a standardized set of human and mouse protein-coding regions supported by expert curation. *Nucleic Acids Res*, **46**, D221-D228.

2. Gene Ontology, C. (2021) The Gene Ontology resource: enriching a GOld mine. *Nucleic Acids Res*, **49**, D325-D334.

3. Caspi, R., Altman, T., Billington, R., Dreher, K., Foerster, H., Fulcher, C.A., Holland, T.A., Keseler, I.M., Kothari, A., Kubo, A. *et al.* (2014) The MetaCyc database of metabolic pathways and enzymes and the BioCyc collection of Pathway/Genome Databases. *Nucleic Acids Res*, **42**, D459-471.

4. Lee, I., Date, S.V., Adai, A.T. and Marcotte, E.M. (2004) A probabilistic functional network of yeast genes. *Science*, **306**, 1555-1558.

5. Hwang, S., Kim, C.Y., Yang, S., Kim, E., Hart, T., Marcotte, E.M. and Lee, I. (2019) HumanNet v2: human gene networks for disease research. *Nucleic Acids Res*, **47**, D573-D580.

6. Lee, I., Blom, U.M., Wang, P.I., Shim, J.E. and Marcotte, E.M. (2011) Prioritizing candidate disease genes by network-based boosting of genome-wide association data. *Genome Res*, **21**, 1109-1121.

7. Orchard, S., Ammari, M., Aranda, B., Breuza, L., Briganti, L., Broackes-Carter, F., Campbell, N.H., Chavali, G., Chen, C., del-Toro, N. *et al.* (2014) The MIntAct project--IntAct as a common curation platform for 11 molecular interaction databases. *Nucleic Acids Res*, **42**, D358-363.

8. Oughtred, R., Rust, J., Chang, C., Breitkreutz, B.J., Stark, C., Willems, A., Boucher, L., Leung, G., Kolas, N., Zhang, F. *et al.* (2021) The BioGRID database: A comprehensive biomedical resource of curated protein, genetic, and chemical interactions. *Protein Sci*, **30**, 187-200.

9. Razick, S., Magklaras, G. and Donaldson, I.M. (2008) iRefIndex: a consolidated protein interaction database with provenance. *BMC Bioinformatics*, **9**, 405.

10. Perez-Riverol, Y., Ternent, T., Koch, M., Barsnes, H., Vrousgou, O., Jupp, S. and Vizcaino, J.A. (2017) OLS Client and OLS Dialog: Open Source Tools to Annotate Public Omics Datasets. *Proteomics*, **17**.

11. Huttlin, E.L., Bruckner, R.J., Navarrete-Perea, J., Cannon, J.R., Baltier, K., Gebreab, F., Gygi, M.P., Thornock, A., Zarraga, G., Tam, S. *et al.* (2021) Dual proteome-scale networks reveal cell-specific remodeling of the human interactome. *Cell*, **184**, 3022-3040 e3028.

12. Kanehisa, M., Furumichi, M., Sato, Y., Ishiguro-Watanabe, M. and Tanabe, M. (2021) KEGG: integrating viruses and cellular organisms. *Nucleic Acids Res*, **49**, D545-D551.

13. Nishimura, D. (2001) BioCarta. *Biotech Software & Internet Report*, **2**, 117-120.

14. Jassal, B., Matthews, L., Viteri, G., Gong, C., Lorente, P., Fabregat, A., Sidiropoulos, K., Cook, J., Gillespie, M., Haw, R. *et al.* (2020) The reactome pathway knowledgebase. *Nucleic Acids Res*, **48**, D498-D503.

15. Meyers, R.M., Bryan, J.G., McFarland, J.M., Weir, B.A., Sizemore, A.E., Xu, H., Dharia, N.V., Montgomery, P.G., Cowley, G.S., Pantel, S. *et al.* (2017) Computational correction of copy number effect improves specificity of CRISPR-Cas9 essentiality screens in cancer cells. *Nat Genet*, **49**, 1779-1784.

16. Kim, E. and Hart, T. (2021) Improved analysis of CRISPR fitness screens and reduced off-target effects with the BAGEL2 gene essentiality classifier. *Genome Med*, **13**, 2.

17. Lawson, K.A., Sousa, C.M., Zhang, X., Kim, E., Akthar, R., Caumanns, J.J., Yao, Y., Mikolajewicz, N., Ross, C., Brown, K.R. *et al.* (2020) Functional genomic landscape of cancer-intrinsic evasion of killing by T cells. *Nature*, **586**, 120-126.

18. Dandekar, T., Snel, B., Huynen, M. and Bork, P. (1998) Conservation of gene order: a fingerprint of proteins that physically interact. *Trends Biochem Sci*, **23**, 324-328.

19. Parks, D.H., Chuvochina, M., Chaumeil, P.A., Rinke, C., Mussig, A.J. and Hugenholtz, P. (2020) A complete domain-to-species taxonomy for Bacteria and Archaea. *Nat Biotechnol*, **38**, 1079-1086.

20. Shin, J., Lee, T., Kim, H. and Lee, I. (2014) Complementarity between distance- and probability-based methods of gene neighbourhood identification for pathway reconstruction. *Mol Biosyst*, **10**, 24-29.

21. Reimand, J., Hui, S., Jain, S., Law, B. and Bader, G.D. (2012) Domain-mediated protein interaction prediction: From genome to network. *FEBS Lett*, **586**, 2751-2763.

22. Blum, M., Chang, H.Y., Chuguransky, S., Grego, T., Kandasaamy, S., Mitchell, A., Nuka, G., Paysan-Lafosse, T., Qureshi, M., Raj, S. *et al.* (2021) The InterPro protein families and domains database: 20 years on. *Nucleic Acids Res*, **49**, D344-D354.

23. Shim, J.E. and Lee, I. (2016) Weighted mutual information analysis substantially improves domain-based functional network models. *Bioinformatics*, **32**, 2824-2830.

24. Szklarczyk, D., Gable, A.L., Nastou, K.C., Lyon, D., Kirsch, R., Pyysalo, S., Doncheva, N.T., Legeay, M., Fang, T., Bork, P. *et al.* (2021) The STRING database in 2021: customizable protein-protein networks, and functional characterization of user-uploaded gene/measurement sets. *Nucleic Acids Res*, **49**, D605-D612.

25. Persson, E., Castresana-Aguirre, M., Buzzao, D., Guala, D. and Sonnhammer, E.L.L. (2021) FunCoup 5: Functional Association Networks in All Domains of Life, Supporting Directed Links and Tissue-Specificity. *J Mol Biol*, **433**, 166835.

26. Franz, M., Rodriguez, H., Lopes, C., Zuberi, K., Montojo, J., Bader, G.D. and Morris, Q. (2018) GeneMANIA update 2018. *Nucleic Acids Res*, **46**, W60-W64.

27. Greene, C.S., Krishnan, A., Wong, A.K., Ricciotti, E., Zelaya, R.A., Himmelstein, D.S., Zhang, R., Hartmann, B.M., Zaslavsky, E., Sealfon, S.C. *et al.* (2015) Understanding multicellular function and disease with human tissue-specific networks. *Nat Genet*, **47**, 569-576.

28. Kamburov, A., Stelzl, U., Lehrach, H. and Herwig, R. (2013) The ConsensusPathDB interaction database: 2013 update. *Nucleic Acids Res*, **41**, D793-800.

29. Huang, J.K., Carlin, D.E., Yu, M.K., Zhang, W., Kreisberg, J.F., Tamayo, P. and Ideker, T. (2018) Systematic Evaluation of Molecular Networks for Discovery of Disease Genes. *Cell Syst*, **6**, 484-495 e485.

30. Welter, D., MacArthur, J., Morales, J., Burdett, T., Hall, P., Junkins, H., Klemm, A., Flicek, P., Manolio, T., Hindorff, L. *et al.* (2014) The NHGRI GWAS Catalog, a curated resource of SNP-trait associations. *Nucleic Acids Res*, **42**, D1001-1006.

31. Pinero, J., Ramirez-Anguita, J.M., Sauch-Pitarch, J., Ronzano, F., Centeno, E., Sanz, F. and Furlong, L.I. (2020) The DisGeNET knowledge platform for disease genomics: 2019 update. *Nucleic Acids Res*, **48**, D845-D855.

32. Initiative, C.-H.G. (2021) Mapping the human genetic architecture of COVID-19. *Nature*.

33. Kuleshov, M.V., Stein, D.J., Clarke, D.J.B., Kropiwnicki, E., Jagodnik, K.M., Bartal, A., Evangelista, J.E., Hom, J., Cheng, M., Bailey, A. *et al.* (2020) The COVID-19 Drug and Gene Set Library. *Patterns (N Y)*, **1**, 100090.

34. Stephenson, E., Reynolds, G., Botting, R.A., Calero-Nieto, F.J., Morgan, M.D., Tuong, Z.K., Bach, K., Sungnak, W., Worlock, K.B., Yoshida, M. *et al.* (2021) Single-cell multi-omics analysis of the immune response in COVID-19. *Nat Med*, **27**, 904-916.

35. Ren, X., Wen, W., Fan, X., Hou, W., Su, B., Cai, P., Li, J., Liu, Y., Tang, F., Zhang, F. *et al.* (2021) COVID-19 immune features revealed by a large-scale single-cell transcriptome atlas. *Cell*, **184**, 1895-1913 e1819.

36. Schulte-Schrepping, J., Reusch, N., Paclik, D., Bassler, K., Schlickeiser, S., Zhang, B., Kramer, B., Krammer, T., Brumhard, S., Bonaguro, L. *et al.* (2020) Severe COVID-19 Is Marked by a Dysregulated Myeloid Cell Compartment. *Cell*, **182**, 1419-1440 e1423.

Supplementary Tables

Supplementary Table 1. Size comparison of gold standard gene pairs and component networks between HumanNet v2 and v3

**Gold standard**					
**HumanNet v2**	**# genes**	**# links**	**HumanNet v3**	**# genes**	**# links**
GO + MetaCyc	5,190	124,950	GO + MetaCyc	8,779	260,962
**Component networks**					
**HumanNet v2**	**# genes**	**# links**	**HumanNet v3**	**# genes**	**# links**
CC	10,303	72,819	CC	18,300	1,081,518
CE	4,052	71,243	GI *	10,478	174,509
CX	6,658	29,481	CX	12,180	81,064
DB	7,512	125,550	DB	8,540	135,327
DP	9,454	45,958	DP	12,700	73,414
GN	2,403	24,862	GN	2,339	97,565
IL	9,077	113,499	IL**	9,001	122,697
PG	1,522	10,888	PG	2,126	16,465
LC	13,790	122,444	PI	17,849	633,460
HT	12,641	78,627			
**Integrated networks**					
**HumanNet v2**	**# genes**	**# links**	**HumanNet v3**	**# genes**	**# links**
PI	15,352	158,499	PI	17,849	633,460
CF	14,739	252,590	-	-	-
FN	17,247	371,502	FN	18,459	977,495
XI	17,303	418,525	FN + Interolog **	18,462	1,033,510
XC	17,790	424,501	XC	18,593	1,125,494
XN	17,929	525,537	XC + Interolog **	18,593	1,175,474

* HumanNet v2-CE corresponds to HumanNet v3-GI.

** These networks are not included in the final set of HumanNet v3.

Supplementary Table 2. Summary of human gene networks compared in this study

Network	# genes	# links
HumanNet v2 (XN)	17,929	525,537
HumanNet-PI	17,849	633,460
HumanNet-FN	18,459	977,495
HumanNet-XC	18,593	1,125,494
STRING (v11.5)	18,073	5,403,129
GeneMania (April 27, 2021)	18,431	11,202,221
CPDB (July 31, 2021)	17,011	536,256
PCNet (August 5, 2021)	18,183	2,670,439
Funcoup (v5.0)	17,388	4,875,873
GIANT (September 25, 2021)	18,446	17,108,785

Supplementary Table 3. Summary of update for gene annotation databases

**Gene set**	**HumanNet v2** (annotation download date)	**HumanNet v3** (annotation download date)
Gene Ontology Biological Process (GOBP)	April 4, 2018	March 8, 2021
GWAS Catalog	September 14, 2018	June 26, 2021
DisGeNET	June 8, 2018	June 23, 2021
DISEASES	June 20, 2018	June 20, 2021
Human Phenotype Ontology (HPO)	March 09, 2018	June 23, 2021

Supplementary Table 4. 43 guide genes used for prediction of COVID-19 genes

ARHGAP27	FDX2	KAT7	OAS2	TAC4
ARL17A	FOXP4	LRRC37A	OAS3	TMEM65
ARL17B	HSD17B14	LRRC37A2	PLEKHA4	TULP2
CCR3	ICAM1	LZTFL1	PLEKHM1	TYK2
CEP97	ICAM3	MAPT	PPP1R15A	WNT3
CRHR1	ICAM4	NSF	RPL24	ZBTB11
CXCR6	ICAM5	NUCB1	SLC6A20	ZGLP1
DLX3	IFNAR2	NXPE3	SPPL2C	
DPP9	KANSL1	OAS1	STH	

Supplementary Figures

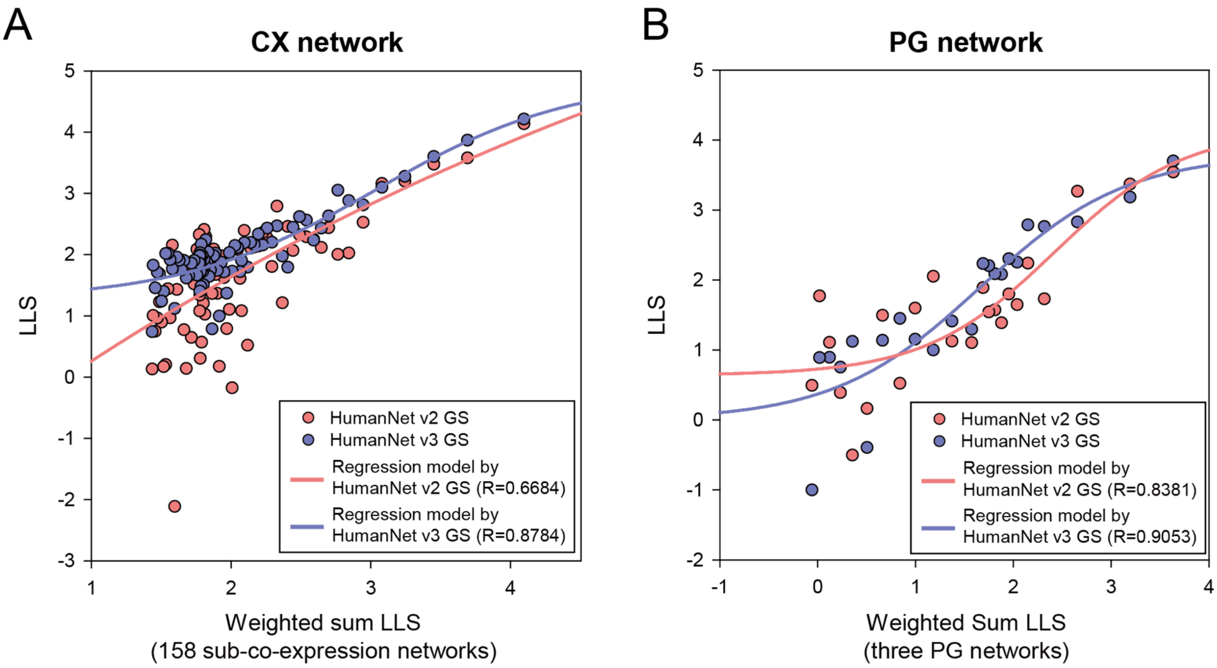

Supplementary Figure 1. Improvement of CX and PG network by retraining with HumanNet v3 gold standard. LLS for every 1,000 links that calculated by HumanNet v3 gold standard (blue) and v2 gold standard (red) in **(A)** CX and **(B)** PG network. Regression models for each network also presented with the same color annotation.

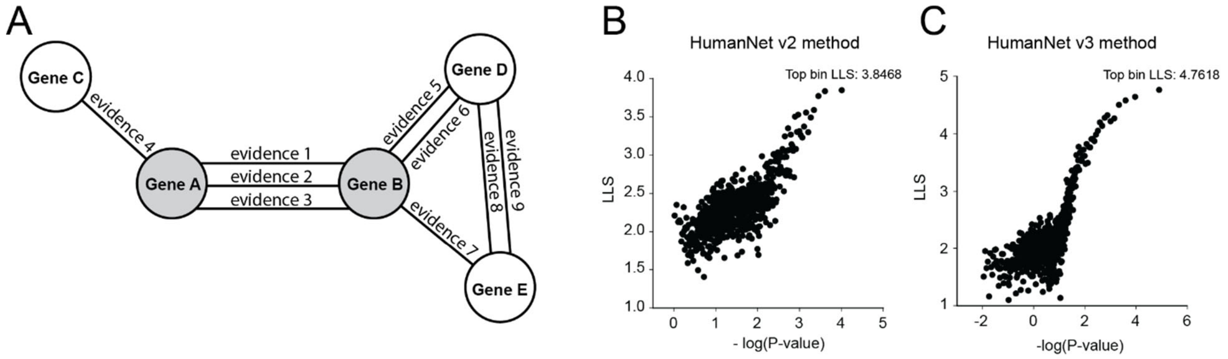

Contingency table for HumanNet v2

# links		
	Gene A	Others
Gene B	1	2
Others	1	1

Contingency table for HumanNet v3

# evidence-links		
	Gene A	Others
Gene B	3	3
Others	1	2

Supplementary Figure 2. Link prioritization in HumanNet v3. **(A)** Examples showing different prioritization method between HumanNet v2 and HumanNet v3. P-value against LLS plot for protein-protein interaction network that prioritized by **(B)** HumanNet v2 method and **(C)** HumanNet v3 method. Each bins represent 1,000 links. LLS for the top 1,000 links is presented.

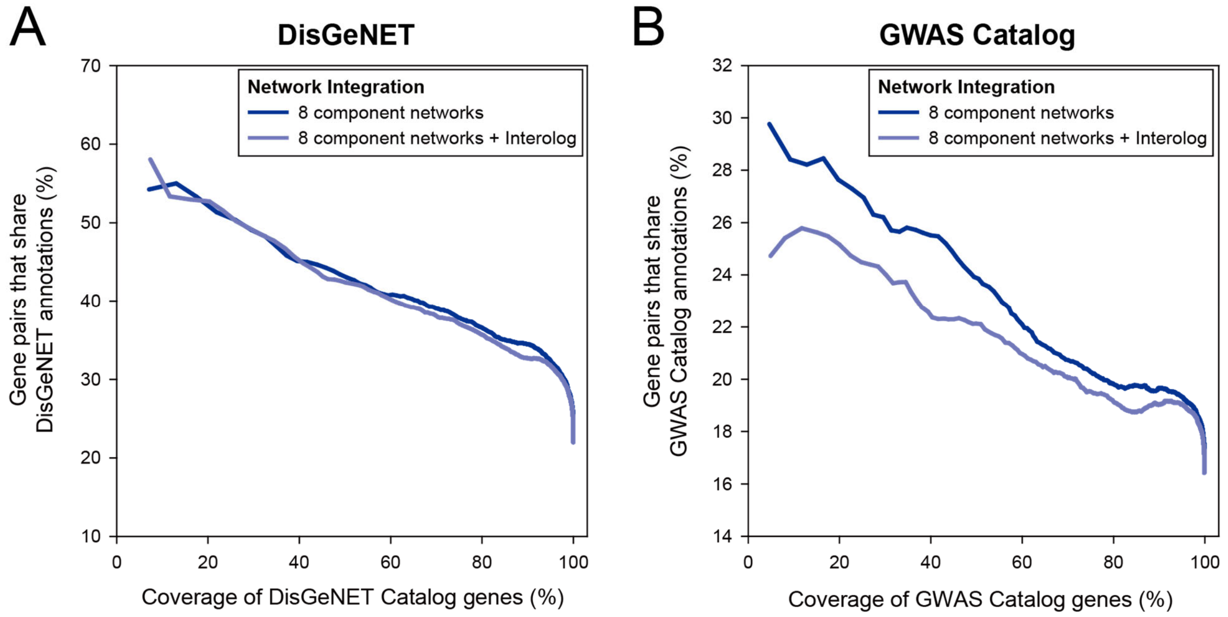

Supplementary Figure 3. Interologs decrease the accuracy of the integrated network for disease gene interactions. The percentage of gene pairs that share disease annotation (y-axis, link precision) according to the DisGeNET **(A)** and GWAS Catalog **(B)** gene coverage (x-axis, gene recall) are cumulatively calculated for every 1,000 links from the top links.
